# Evaluation of single nucleotide polymorphisms in microRNAs (hsa-miR-196a2 rs11614913 C/T) from Brazilian women with breast cancer

**DOI:** 10.1186/1471-2350-13-119

**Published:** 2012-12-10

**Authors:** José Juvenal Linhares, Marcos Azevedo, Adalberto Abraão Siufi, Cristina Valleta de Carvalho, Maria Del Carmen Garcia Molina Wolgien, Emmanuelle Coelho Noronha, Tatiana Carvalho de Souza Bonetti, Ismael Dale Cotrim Guerreiro da Silva

**Affiliations:** 1 Department of Gynecology and Obstetrics, Federal University of Ceará (Campus Sobral), Av. Humberto Lopes, 200, Junco, Sobral, Ceará, CEP: 62022-304, Brazil; 2 Molecular Gynecology Laboratory, Department of Gynecology, Federal University of São Paulo, Rua Pedro de Toledo, 781 – 4o. Floor, Vila Clementino, São Paulo-SP, CEP: 04039-032, Brazil; 3 Biology Department, Centro Universitário Fundação Santo André, São Paulo-SP, Brazil; 4 Reproductive Biology Research Laboratory, Division of Reproductive Endocrinology and Infertility, Department of Obstetrics and Gynecology, University of South Florida – USF, MDC 2147, 12901 Bruce B Downs Blvd, Tampa, FL, 33612, USA

**Keywords:** Breast, Cancer, Polymorphisms, MicroRNAs

## Abstract

**Background:**

Emerging evidence has shown that miRNAs are involved in human carcinogenesis as tumor suppressors or oncogenes. Single nucleotide polymorphisms (SNPs) located in pre-miRNAs may affect the processing and therefore, influence the expression of mature miRNAs. Previous studies generated conflicting results when reporting association between the hsa-miR-196a2 rs11614913 common polymorphism and breast cancer.

**Methods:**

This study evaluated the hsa-miR-196a2 rs11614913 SNP in 388 breast cancer cases and 388 controls in Brazilian women. Polymorphism was determined by real-time PCR; control and experimental groups were compared through statistical analysis using the X^2^ or Fisher’s exact tests.

**Results:**

The analysis of the SNPs frequencies showed a significant difference between the groups (BC and CT) in regards to genotype distribution (χ^2^: p = 0.024); the homozygous variant (CC) was more frequent in the CT than in the BC group (p = 0.009). The presence of the hsa-miR-196a2 rs11614913 C/T polymorphism was not associated with histological grades (p = 0.522), axillary lymph node positive status (p = 0.805), or clinical stage (p = 0.670) among the breast cancer patients.

**Conclusions:**

The results of this study indicated that the CC polymorphic genotype is associated with a decreased risk of BC and the presence of the T allele was significantly associated with an increased risk of BC.

## Background

MiRNAs represent a recently discovered class of small regulatory RNAs that influence the stability and efficiency of target mRNAs and are implicated in a growing number of biological processes including neoplasms. Recent studies have demonstrated the involvement of these regulatory molecules in the development of breast cancer (BC) [1].

Zhang et al. compared the expression patterns of miRNAs between healthy and BC mammary tissues and observed, preliminarily, that various miRNAs are expressed in different ways in BC. This observation lead to the hypothesis that expression profiles of miRNAs could be correlated with special tumor phenotypes or associated with tumor suppression or tumorigenesis [2]. Iorio et al., using microarray analysis, demonstrated that aberrant expression of miRNAs is associated with human BC [3].

MiRNAs have an enormous potential as biomarkers of BC because their expression is known to be aberrant in cancer with pathognomonic profiles, that is, related to specific tissues; in addition, they are notably stable molecules that remain well preserved in formalin, paraffin-embedded tissues, and fresh or frozen tissues [4,5].

A strong association between miRNA polymorphisms and risk of cancer has been established and, thus provides supporting pathways for the investigation of molecular mechanisms involved in the development of cancer. The loss or gain of function of particular miRNAs are also involved in events during the genesis of various types of cancer, including BC [6]. For example, hsa-mir-10B has been identified as an initiator of invasion and metastasis in BC [7], where expression patterns are altered in human BC [3,8].

Because BC is an estrogen-dependent tumor, mechanisms involving the estrogen receptor have been considered in studies addressing the molecular understanding of this disease. Estrogen receptors have been recently shown to be regulated by miRNAs as well as other cofactors. miRNAs are regulatory RNA molecules that have been foremost in the molecular investigation of many diseases and cellular processes. They exert their effect directly on target genes resulting in transcriptional repression or degradation of the target sequence [9,10].

The hsa-miR-196a2 rs11614913 polymorphism, located on chromosome 12 (12q13.13), appears to regulate the Hox gene in a subgroup of the homeobox genes. This subgroup controls the development and positional differentiation of cells in the embryo. Thus, the prediction of the miRNAs that would target homeobox genes (together with genes that have an important function in development through the control of the parts of the embryo that will develop into organs and specific tissues) seem to be an especially attractive area for the investigation of breast cancer development [11].

The modulation of the p53 gene expression is controled by HOX proteins that bind to its promoter region [12,13]. In addition, the expression of these proteins can lead to inhibition of p53-dependent apoptosis in BC cells. Therefore, the decreased gene expression in mammary tumors can act on p53, firstly through the promotion of genome instability, and secondly through opposing mechanisms that lead to apoptosis there after [10,12,14].

In a case–control study with Chinese women with breast cancer, the genotype variant of hsa-miR-196a2 rs11614913 was associated with a significantly increased risk of BC [15].That study provided the first evidence that SNPs, common in miRNAs, could be utilized as candidate biomarkers for BC susceptibility.

A recent case–control study with Italian and German women that evaluated miRNAs polymorphisms showed that the hsa-miR-196a2 rs11614913 genotype was not associated with increased risk of BC [16].

The correlations between the hsa-miR-196a2 rs11614913 C/T SNP and development of breast cancer are controversially reported in the literature. Moreover, there are no relevant studies in South American populations reporting on this subject. Hence, this study assessed the presence of hsa-miR-196a2 rs11614913 polymorphisms in microRNA sequences from Brazilian women with and without breast cancer and its association with breast cancer.

## Methods

### Subjects

The Research Ethics Committee from the Federal University of Sao Paulo (No. 0211/08) approved this study; signed informed consents were obtained from all participants before the study start. The study included 388 women diagnosed between April 2004 and July 2010 with invasive ductal breast carcinoma (BC group) in the Mastology Division of the “Francisco Morato Oliveira” Hospital (HSPE-FMO) and Federal University of São Paulo (UNIFESP-EPM) in Brazil. The diagnosis of breast carcinoma was reached through clinical and radiological examinations (mammography and/or ultrasonography) and subsequently confirmed by the histopathological assessment of biopsies. Peripheral blood samples were collected regardless of the clinical stage or previous treatment. Pregnant women or patients under hormonal therapy (HT) were excluded from the study.

The control group (CT group) included samples of oral mucosal scrapings obtained from 388 women without breast cancer attending the Menopause and Gynecology Clinic for routine consultations at the same institutions mentioned above. These patients were also screened for breast cancer through clinical and radiological examinations (mammography and/or ultrasound); patients under hormonal therapy (HT), pregnant, or with a prior history of breast biopsy were excluded. The oral samples from these patients were collected using a Cytobrush® and stored in dry tubes.

Data from all patients were collected in a standardized form containing information about age, race (Caucasianornon-Caucasian), age at menarche (under or over 12 years of age), pre or post-menopausal status, family history of breast cancer (first-degree relative: mother, sister, or daughter), the habit of smoking, cancer clinical stage, axillary lymph node status, and Scarff-Bloom-Richardson (SBR) histological grade.

### Genotyping

DNA was extracted from blood and oral mucosal scrapping samples according to the protocol for the GFX® kit for cells, tissues, and blood (Amersham-Pharmacia).

The hsa-miR-196a2 rs11614913 C/T SNP was genotyped using the *Custom TaqMan*® *SNP Genotyping Assays* (Applied Biosystems, ID C_31185852_10). Between 1 and 20 ng of DNA was used per PCR reaction performed in duplicates at the final volume of 20 ul using the ABI 7500 Real-Time PCR detection System (*Applied Biosystems*®).

The cycling conditions were 50°C for 2 min, 95°C for 10 min, 40 cycles of at 92°C for 15 s, and annealing/extension at 60°C for 1 min. The data were analyzed using the *SDS Software v*. *1*.*3*.*1*.

### Statistical analysis

The outcomes were analyzed taking into consideration the patients’ characteristics assessed in the standardized form used for data collection. First-degree relatives such as mother, sisters, or daughters were considered in the assessment of family history of breast cancer.

The demographic characteristics of the groups were compared by the Student's t-test for continuous variables and the qui-squared test (χ2) for nominal variables. The Pearson’s chi-square test was used to assess the correlation between variables; multiple linear and logistic regression models were used to evaluate associations between genotypes, alleles, and occurrence of breast cancer. The CC genotype was considered as the reference for comparisons with the TC (heterozygote) and TT (homozygote) variants. The sample power was calculated based on the proportion of patients with at least one mutated allele (TC or TT). The sample power in this study was 0.77 considering an alpha error = 0.05. The SPSS 15 statistical software was used for data analyses and a p value ≤ 0.05 was considered statistically significant.

## Results

The demographic characteristics and risk factors associated with the BC and CT groups are shown in Table 1. The BC group presented an average age that was 2.6 years older than the CT group and a higher percentage of non-Caucasian patients. Axillary lymph node positive status was observed in 45.8% of the patients in the BC group; breast cancer histological grades and clinical stages are shown in Table 1.

**Table 1:** Summary of demographic characteristics and risk factors for breast cancer development

**VARIABLE**	**CT group (n = 388)**	**BC group (n = 388)**	***p *value**
Age in years (mean ± SD)	55.1 ± 10.3	57.7 ± 12.6	0.008
Age at menarche in years (mean ± SD)	12.9 ± 1.6	13.0 ± 1.6	0.700
Race			
Caucasian	19.4%	16.2%	<0.001
Non-Caucasian	70.6%	83.8%	
Patients at Menopause	66.8%	63.9%	0.225
Habit of smoking	26.3%	22.4%	0.240
First-degree family history of breast cancer	16.7%	18.0%	0.635
Histological Grade			
1	----	24.5%	----
2	----	47.9%	----
3	----	27.6%	----
Clinical stages			
1	----	34.5%	----
2	----	43.8%	----
3	----	16.0%	----
4	----	5.7%	----

The genotype distribution in the CT and BC groups are shown in Table 2; 100% concordance was observed between the results from the samples’ duplicates. The analysis of the frequencies showed a significant difference in genotype distribution between groups (BC and CT) (χ^2^: p = 0.024); the homozygous variant (CC) was more frequent in the CT group than in the BC group (p = 0.009).

**Table 2:** Genotype distribution in the CT and BC groups

**GROUPS**		**Genotype**			**HWE**
		**CC**	**TC**	**TT**	
CT	Count	127	165	96	0.004
	% within Group	32.7%	42.5%	24.7%	
BC	Count	94	177	117	0.096
	% within Group	24.2%	45.6%	30.2%	

HWE: Hardy-Weinberg Equilibrium.χ^2^: p = 0.024.

The Brazilian population is characterized by a mixture of races, which justifies the HWE deviation in the distribution of genetic variants. Thus, the HWE was calculated based on Caucasian or non-Caucasian races in both groups, the control group (p = 0.008 and 0.264), and BC group (p = 0.112 and p = 0.723). The control Caucasian group presented an unbalanced distribution of variants (Table 3).

**Table 3:** Genotype distribution in the CT and BC groups based on races

**GROUPS**		**Genotype**			**HWE**
		**CC**	**TC**	**TT**	
CT Caucasian	Count	94	114	66	0.008
	% within Group	34.3%	41.6%	24.1%	
CT non-Caucasian	Count	33	51	30	0.264
	% within Group	29.0%	44.7%	26.3%	
BC Caucasian	Count	83	148	94	0.112
	% within Group	25.5%	45.6%	28.9%	
BC non-Caucasian	Count	11	29	23	0.723
	% within Group	17.5%	46.0%	36.5%	

HWE: Hardy-Weinberg Equilibrium.

A multiple logistic regression analysis was conducted to evaluate the association of the hsa-miR-196a2 rs11614913 C/T polymorphism adjusted for interfering variables as age, race, menarche age, menopausal status, smoking habits, and first-degree breast cancer family history. The presence of the TC or TT variants increased the risk of breast cancer in 50% when adjusted for the confounding variables (Table 4).

**Table 4:** Multiple logistic regression analysis adjusted for the interfering variables to evaluate the association between polymorphism and occurrence of BC

	**Constant**	**SE**	**OR**	***P***
Presence of TC or TT variant	0.406	0.165	1.501	0.014
Age (years)	0.023	0.008	1.024	0.003
Non-Caucasian race	−0.786	0.180	0.456	0.000
Age at Menarche (years)	0.021	0.047	1.021	0.660
Menopause (yes or no)	−0.523	0.194	0.593	0.007
Smoking (yes or no)	−0.192	0.190	0.825	0.311
BC Family history (yes or no)	−0.291	0.187	0.748	0.119
Constant	−1.244	0.733	0.288	0.090

SE: standard error. OR: odds ratio.

The presence of the hsa-miR-196a2 rs11614913 C/T polymorphism was not associated with histological grade (p = 0.522), axillary lymph node positive status (p = 0.805), or clinical stage (p = 0.670) among breast cancer patients. On the other hand, histological grade was negatively associated with age (Pearson correlation: r = −0.106; p = 0.037) and positively associated with smoking (Pearson correlation: r = 0.106; p = 0.038). The axillary lymph node positive status was positively associated with a family history of breast cancer (Pearson correlation: r = 0.190; p < 0.001). The clinical stage of breast cancer was not associated with the hsa-miR-196a2 rs11614913 C/T polymorphism or demographic variables.

## Discussion

The growing interest in evaluating the association between the hsa-miR-196a2 rs11614913 SNP and development of BC [3,4,15-17], along with the lack of studies in Latin America on this subject, were the foundations for this study.

Many associations between miRNAs and occurrence of BC have been demonstrated experimentally. The understanding of the mechanisms involved in these associations could establish the use of these markers (miRNAs) as powerful tools for the prevention and treatment of BC [9].

MiRNAs are involved in critical biological processes including development, differentiation, apoptosis, and proliferation. Genetic polymorphisms of miRNAs (SNPs) can potentially influence the processing or selection of miRNA targets. The association between hsa-miR-196a2 rs11614913 SNPs and susceptibility to develop breast cancer have been evaluated by several recent studies [15,16,18].

Considering the important role of hsa-miR-196a2 rs11614913 in the carcinogenic process of breast cancer, the present study evaluated the distribution of this genotype and estimated the risk of the development of BC in Brazilian women.

Predominance of the TC genotype (heterozygote) was observed in the subjects in the study and corroborated the data reported in the literature [15,16,18]. The analyzed groups in this study were representative of a miscegenated population (Brazilians) and cannot be compared with other studied populations. The predominant allele distribution observed in this population did not diverge from that reported in the literature, despite the miscegenation, suggesting that the heterozygous form is the most prevalent worldwide.

Hu et al. observed an increased risk of breast cancer associated to the TC/CC genotypes with OR of 1.23 (1.02–1.48) when studying an Asian population [15]. Gao et al. conducted a meta-analysis of the hsa-miR-196a2 distribution that suggested that women with the CC polymorphic genotype had an increased risk of breast cancer with OR of 1.30 (1.01-1.68) [19]. Nevertheless, the results from these studies remain conflicting and inconclusive [19]. Conversely, Catucci et al. did not identify statistical correlations between the presence of hsa-miR-196a2 rs11614913 in women with breast cancer and controls in German and Italian populations [16]. The present study produced different results showing an association between the presence of the T (TC/TT) allele and increased risk of BC with OR of 1.52 (1.11-2.08); in addition, the results indicate that the CC (mutant homozygous) polymorphic genotype acted as a protective factor with OR of 0.61 (0.42-0.89). The discordant results could be suggestive of the extensive miscegenation between different ethnic groups in the Brazilian population, which could lead to increased genetic variability. In a meta-analysis including different populations, where a statistical rearrangement was performed, the tendency of the hsa-miR-196a2 rs11614913 SNP to act as a protective factor against breast cancer was observed with OR of 0.83(0.77–1.03) and p = 0.07; these results corroborate the results observed in the meta-analysis [16]. Wang et al. observed that the increased risk of BC associated with the CC genotype was present in Chinese and Indian women but not in Caucasian women; the latter being closer to the Brazilian population than the Asian populations [20].

## Conclusions

The results obtained in this study relative to the hsa-miR-196a2 rs11614913 SNP in the Brazilian population indicated that the CC polymorphic genotype is associated with a decreased risk of BC, and the presence of the T allele is significantly associated with an increased risk of BC. Further studies with larger sample sizes and in populations with similar ethnic background could contribute in supporting the results presented in the current study.

## Abbreviations

BC: Breast cancer; OR: Odds ratio; CI: Confidence interval; HSPE-FMO: “Francisco morato oliveira” hospital; UNIFESP-EPM: Federal university of são paulo, brazil; SBR: Scarff-bloom-richardson.

## Competing interests

The authors declare that they have no competing interests.

## Authors’ contributions

JJL participated in the design of the study, statistical analysis, and manuscript preparation. MAJ and ECN analyzed the data from the real time PCR assays. AAS, CVC, and MDCGMW participated in sample collection. TCSB performed the statistical analyses. IDCGS participated in the design of the study and manuscript preparation. All authors read and approved the final manuscript.

## Pre-publication history

The pre-publication history for this paper can be accessed here:

http://www.biomedcentral.com/1471-2350/13/119/prepub
